# 

**Published:** 1856-12

**Authors:** 


COMMUNICATIONS FOR PUBLICATION TO BE ADDRESSED TO 'EDITORIAL COMMITTEE.'

### IOWA MEDICAL JOURNAL.

CONDUCTED BY THE FACULTY OF THE

COLLEGE OF PHYSICIANS AND SURGEONS

OF THE

IOWA UNIVERSITY.

PUBLISHED EVERY ALTERNATE MONTH.

TERMS—TWO DOLLARS IN ADVANCE.

VOL. III. NOVEMBER AND DECEMBER, 1856. NO. 6.

EDITORIAL COMMITTEE,

D. L. M'GUGIN, M. D.

JNO. R. ALLEN, M. D.

SMITHSONIAN INSTITUTION

16297

FINANCE COMMITTEE.

J. C. HUGHES, M. D.

KEOKUK, IOWA.

PRINTED AT THE POST BOOK AND JOB OFFICE.

BUSINESS COMMUNICATIONS TO THE 'FINANCE COMMITTEE.'

